# Supplementary material for: Electrocorticographic evidence of a common neurocognitive sequence for mentalizing about the self and others
Source: Nat Commun. 2022 Apr 8;13:1919. doi: 10.1038/s41467-022-29510-2 (PMC8993891; doi:10.1038/s41467-022-29510-2)
Supplement: Supplementary file 1 — Supplementary Information [file 41467_2022_29510_MOESM1_ESM.pdf]

Electrocorticographic evidence of a common neurocognitive sequence for mentalizing about the self and others

Supplementary Information

Kevin M. Tan[1], Amy L. Daitch[2], Pedro Pinheiro-Chagas[2], Kieran C.R. Fox[2,3], Josef Parvizi[2,3], Matthew D. Lieberman[1]

[1] Social Cognitive Neuroscience Laboratory, Department of Psychology, University of California, Los Angeles, CA, USA

[2] Laboratory of Behavioral and Cognitive Neuroscience, Department of Neurology and Neurological Sciences, Stanford University, CA, USA

[3] School of Medicine, Stanford University, CA, USA

Correspondence: Matthew D. Lieberman (lieber@ucla.edu) & Kevin M. Tan (kevmtan@ucla.edu)

Contents

Supplementary Methods: Controlling for stimulus visual dissimilarity 2-4

Supplementary Figure 1: Visual dissimilarity of task stimuli 2

Supplementary Figure 2: Within-participant analytic pipeline 5

Supplementary Figure 3: Trialwise propagation of activation onsets across ROIs 6

Supplementary Figure 4: Behavioral results 7

Supplementary Figure 5: ROI activation latencies using behavior-matched trials 8

Supplementary Figure 6: Expanded lateralized views of Fig. 3a 9

Supplementary Figure 7: Expanded lateralized views of Fig. 3c 10

Supplementary Figure 8: Expanded lateralized views of Fig. 4a 11

Supplementary Figure 9: Expanded lateralized views of Fig. 5a 12

Supplementary Table 1: Aggregate ROI encoding of visual and mentalizing-related effects 4

Supplementary Table 2: Participant demographics and characteristics 13

Supplementary Table 3: Summary statistics of single-trial HFB metrics 14

Supplementary Table 4: Stimulus prompts 15

Supplementary References 16

Supplementary Methods: Controlling for stimulus visual dissimilarity

Computer vision methods

To account for visual dissimilarity between task stimuli (alphanumeric prompts; see Fig. 1a & Supplementary Table 4), we used a popular computer vision model for object recognition: the Jarrett Hierarchical Model (JHM; Jarrett et al., 2009[1]). We chose JHM due to its high proficiency in alphanumeric character recognition (99.47% accuracy on 50,000 alphanumeric images), along with its biologically-inspired design (based on the ventral visual stream hierarchy).

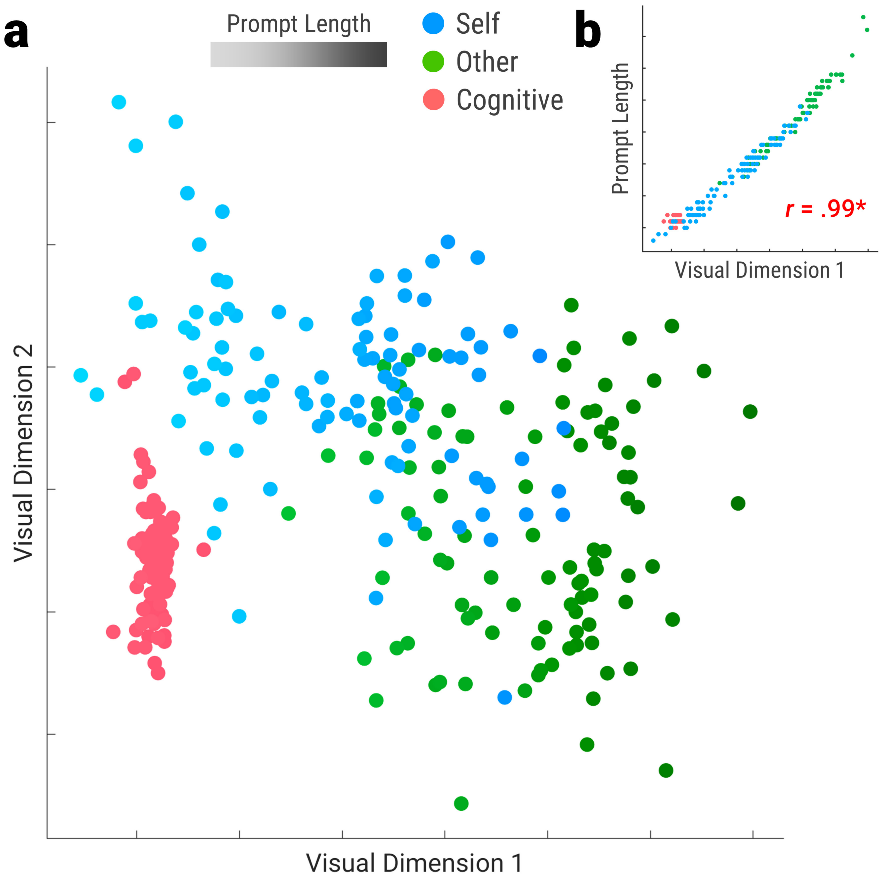

Supplementary Figure 1: Visual dissimilarity of task stimuli using the Jarrett Hierarchical Model (JHM). **a)** Multidimensional scaling of JHM stimulus representations into two visual dimensions (VDs). **b)** Correlation between VD1 and prompt length (number of characters): r_{293}=.991, p<2.23e-308. Source data are provided as a Source Data file.

First, we rendered all stimulus prompts in the manner they were presented to participants (white text on black screen, 1200x800 pixels; Fig. 1a). Prompt renders were then analyzed by JHM, producing output feature maps of size 61x41 per prompt. To quantify visual dissimilarity across stimuli, feature maps of each prompt were compared through representational similarity analysis[2]. The resulting dissimilarity matrix was collapsed into two visual dimensions (VD1 and VD2) using nonmetric multidimensional scaling with Kruskal's normalized stress1 criterion[3]. Note that VDs were purely data-driven; all JHM features were used with no manual pre-selection or weighting.

To confirm whether VD1 and VD2 reasonably represented task prompts, we plotted each stimulus in the Cartesian space formed by the two VDs (Supplementary Fig. 1a), revealing the expected visual dissimilarity across experimental conditions. To confirm that VDs represented prompt length, the primary visual feature of interest, we correlated VDs with the number of characters in each stimulus (Supplementary Fig. 1b). VD1 had a very robust correlation with prompt length (r_{293}=.991, p<2.23e-308), while the correlation with VD2 was nonsignificant (r_{293}=-.019, p=0.747). As such, we surmise that VD1 represents prompt length, while VD2 represents remaining visual features.

Statistical methods

We aimed to reveal which regions-of-interest (ROIs) encoded the VDs while controlling for mentalizing-related effects (mentalizing type, RT_{Behav}, and $Choice_{Behav}$; Supplementary Table 1). To this end, we analyzed single-trial metrics of high-frequency broadband activity (HFB; see Fig. 1de) from mentalizing-active sites (see

Fig. 3ab) using linear mixed-effects models (LMEMs). VDs were represented as fixed and random effects, while mentalizing-related effects were represented as random effects. LMEMs were nested within Site and Participant. To minimize estimation bias, LMEMs used restricted maximum likelihood estimators. To account for full data dependence structure with reduced bias and assumptions, LMEMs used unconstrained variance-covariance matrices with log-Cholesky parametrization. To account for heterogenous variances across LMEM terms, Satterthwaite approximation for degrees of freedom (effective *DF*) was used. To rectify violations of assumptions and overparameterization in LMEMs, we evaluated objective function Hessian matrices and ensured positive definiteness (see *Statistics* in Main Methods).

To control for stimulus visual dissimilarity in the main aggregate ROI analyses (Figs. 4de, 5c-e & Table 1), VD1 and VD2 were used as 'nuisance' random effects in LMEMs as specified in the main manuscript (see *Statistics* and *Aggregate ROI analyses* in Main Methods for further details).

Neuronal encoding of mentalistic and visual features

When examining ROIs for visual encoding while controlling for mentalizing-related effects, we found that visual cortex had more numerous significant VD associations than other ROIs (Supplementary Table 1). Within the default mode network (DMN), some significant VD associations were found in temporoparietal DMN ROIs (tpDMN): temporoparietal junction (TPJ), anterior temporal lobe (ATL), and posteromedial cortex (PMC). As expected, visual encoding did not extend to the medial prefrontal cortices (mPFC) of DMN, as all VD effects were nonsignificant in mPFC ROIs: anteromedial prefrontal cortex (amPFC), dorsomedial prefrontal cortex (dmPFC), and ventromedial prefrontal cortex (vmPFC).

After controlling for VDs in the main aggregate ROI analyses, mentalizing-related effects (mentalizing type, RT_{Behav}, and $Choice_{Behav}$) were still significant in all DMN ROIs (Figs. 4de, 5c-e & Supplementary Table 1). However, in visual cortex, the only notable mentalizing-related effect – marked self/other differences in peak power – became nonsignificant (Fig. 5e).

Taken together, DMN encoding of mentalistic features appears distinct from visual features such as prompt length. In contrast, visual cortex encoding appears primarily driven by visual features rather than mentalizing *per se*. Intriguingly, tpDMN ROIs significantly encoded both mentalistic and visual features – even when controlling for each other – using common HFB metrics (Supplementary Table 1), indicating that tpDMN regions encode mentalistic and visual features in a simultaneous yet dissociable manner. Indeed, numerous neuroimaging studies report simultaneous visual and semantic encoding in tpDMN regions[4–6]. Meanwhile, we found that mPFC ROIs produced numerous significant mentalizing-related effects yet no significant VD effects (Supplementary Table 1), adding further evidence that mPFC regions are situated at the highest levels of the cortical hierarchy, furthest removed from concrete sensorimotor processing. As such, mPFC appears most specialized for highly abstract mentalistic processing – though we do not claim that mPFC is *only* specialized for mentalizing (see Main Discussion).

Supplementary Table 1: Aggregate ROI encoding of visual and mentalistic features. Regions-of-interest (ROI) are defined in Fig. 1c. Significant *p*-values are bolded (*p*<.05, uncorrected, two-tailed). Linear mixed-effect modeling (LMEM) was used to analyze single-trial activation metrics (see Fig. 1e) from mentalizing-active ROI sites (see Fig. 3a) during mentalizing. To account for site- and participant-related heterogeneity, LMEMs were nested within Site and Participant. Separate LMEMs were used per ROI and HFB metric. **VD1** and **VD2** indicate visual dimensions extracted from computer vision representations of task stimuli (see Supplementary Fig. 1). Each VD effect was controlled for all other effects shown here. **Type**$_{\textbf{Other-Self}}$ indicates mentalizing type differences, controlled for RT$_{\text{Behav}}$ and VDs (Fig. 5c-e). **RT**$_{\textbf{Behav}}$ indicates behavioral response time effects, controlled for behavioral response choices and VDs (Fig. 4de). **Choice**$_{\textbf{True-False}}$ indicates behavioral response choice differences, controlled for RT$_{\text{Behav}}$ and VDs (see Fig. 1a for task). Note: non-VD results are copied from Table 1. *Abbreviations*: ROI = region-of-interest, HFB = high-frequency broadband (70-180 Hz), ms = millisecond, z = z-score, *b* = slope estimate, *SE* = standard error of *b*, Visual = visual cortex, ATL = anterior temporal lobe, TPJ = temporoparietal junction, PMC = posteromedial cortex, amPFC = anteromedial prefrontal cortex, dmPFC = dorsomedial prefrontal cortex, vmPFC = ventromedial prefrontal cortex. Source data are provided as a Source Data file.

	**Onset (ms)**			**Peak (ms)**			**Offset (ms)**			**Duration (ms)**			**Peak Power (z)**		
**VD1**	*b*	*SE*	*p*	*b*	*SE*	*p*	*b*	*SE*	*p*	*b*	*SE*	*p*	*b*	*SE*	*p*
Visual	-.220	.090	**.015**	.436	.027	**<.001**	10.646	.281	**<.001**	1.389	.442	**.002**	1.827	.595	**.002**
TPJ	.339	.427	.428	.834	.845	.323	2.469	.522	**<.001**	1.817	.598	**.002**	2.096	.627	**.001**
ATL	.008	.453	.986	.065	.561	.908	.888	.649	.171	1.252	.539	**.020**	1.052	.496	**.034**
PMC	-.186	.277	.502	.294	.518	.571	1.184	.412	**.004**	1.195	.636	.060	2.824	.617	**<.001**
amPFC	.014	.661	.983	1.431	1.344	.287	.803	.564	.155	3.396	5.351	.526	.582	.901	.518
dmPFC	.247	.379	.515	1.178	.760	.121	.807	.509	.113	3.778	3.255	.246	1.693	.967	.080
vmPFC	.402	.693	.562	.387	1.199	.747	.193	.781	.805	1.034	1.288	.422	1.682	1.190	.158
**VD2**	*b*	*SE*	*p*	*b*	*SE*	*p*	*b*	*SE*	*p*	*b*	*SE*	*p*	*b*	*SE*	*p*
Visual	.470	.564	.405	-2.549	1.056	**.016**	-25.244	1.242	**<.001**	-5.589	1.857	**.003**	-3.262	2.708	.228
TPJ	.618	1.622	.703	2.386	3.608	.509	6.472	2.352	**.006**	-1.280	2.412	.596	2.142	2.844	.451
ATL	-1.971	1.838	.284	-2.051	3.255	.529	-44.613	2.347	**<.001**	-5.561	2.601	**.033**	-4.907	2.499	**.050**
PMC	.534	2.127	.802	-4.644	3.391	.171	-2.883	2.495	.248	-.014	2.846	.996	-5.362	2.921	.066
amPFC	-2.978	2.346	.205	-10.103	8.146	.215	-4.522	2.600	.082	-.222	3.668	.952	-2.873	4.494	.523
dmPFC	.424	1.989	.831	-5.227	3.397	.124	-3.005	1.997	.133	4.116	2.432	.091	2.388	2.971	.422
vmPFC	1.902	3.209	.553	-.519	5.712	.928	-3.712	4.161	.373	-5.678	5.423	.295	-13.799	9.106	.130
**Type** $_{\textbf{Other-Self}}$	*b*	*SE*	*p*	*b*	*SE*	*p*	*b*	*SE*	*p*	*b*	*SE*	*p*	*b*	*SE*	*p*
Visual	-4	3	.230	2	7	.731	10	14	.456	7	19	.718	.025	.013	.058
TPJ	1	14	.962	11	46	.808	2	28	.951	4	28	.886	.002	.017	.901
ATL	16	18	.376	80	30	**.007**	58	26	**.026**	71	28	**.013**	.021	.012	.091
PMC	-12	7	.088	71	15	**<.001**	112	18	**<.001**	101	40	**.011**	.251	.045	**<.001**
amPFC	34	28	.230	182	30	**<.001**	151	13	**<.001**	158	21	**<.001**	.013	.074	.857
dmPFC	20	18	.279	189	45	**<.001**	130	9	**<.001**	130	20	**<.001**	.023	.041	.569
vmPFC	21	37	.571	203	63	**.001**	160	18	**<.001**	193	52	**<.001**	.147	.154	.341
**RT** $_{\textbf{Behav}}$	*b*	*SE*	*p*	*b*	*SE*	*p*	*b*	*SE*	*p*	*b*	*SE*	*p*	*b*	*SE*	*p*
Visual	.004	.002	**.021**	.006	.004	.143	.897	.006	**<.001**	.239	.015	**<.001**	.014	.008	.106
TPJ	.025	.005	**<.001**	.292	.023	**<.001**	.904	.014	**<.001**	.143	.016	**<.001**	.085	.018	**<.001**
ATL	.009	.008	.272	.270	.023	**<.001**	.772	.015	**<.001**	.096	.011	**<.001**	.020	.012	.086
PMC	.017	.007	**.012**	.336	.493	**<.001**	.853	.023	**<.001**	.209	.012	**<.001**	.033	.018	.060
amPFC	.041	.015	**.006**	.428	.024	**<.001**	.970	.008	**<.001**	.158	.018	**<.001**	.037	.021	.076
dmPFC	.038	.008	**<.001**	.426	.021	**<.001**	.987	.007	**<.001**	.183	.015	**<.001**	.086	.013	**<.001**
vmPFC	.010	.019	.604	.407	.105	**<.001**	.987	.015	**<.001**	.197	.041	**<.001**	.058	.090	.521
**Choice** $_{\textbf{True-False}}$	*b*	*SE*	*p*	*b*	*SE*	*p*	*b*	*SE*	*p*	*b*	*SE*	*p*	*b*	*SE*	*p*
Visual	0	3	.991	-21	12	.079	-11	8	.141	-21	14	.138	-.025	.013	.058
TPJ	-2	14	.911	-14	28	.616	-20	21	.347	-16	25	.509	.021	.031	.504
ATL	-9	20	.651	-21	34	.548	-12	21	.563	-21	20	.278	-.017	.022	.447
PMC	-10	10	.355	-5	16	.765	-3	15	.848	-3	20	.869	-.010	.023	.650
amPFC	19	25	.463	-43	34	.206	-13	17	.451	-36	29	.221	-.021	.028	.444
dmPFC	16	15	.312	-27	28	.334	-21	9	**.020**	-27	13	**.040**	-.076	.030	**.011**
vmPFC	26	44	.562	-50	53	.350	-10	25	.686	-50	48	.300	-.006	.041	.875

***N* per ROI (trials x sites):** Visual = 8453; TPJ = 3189; ATL = 3046; PMC = 4301; amPFC = 2012; dmPFC = 3438; vmPFC = 1060

Supplementary Figure 2. Within-participant analytic pipeline using Participant 5. Exemplar ROI sites are indicated by arrows in Panel C. **a)** Heatmaps of high-frequency broadband power (HFB; 70-180 Hz) within each timepoint and trial (z-scored). Black areas indicate timepoints discarded due to presentation of the next trial's stimulus. **b)** Trial-averaged timecourses of evoked HFB responses (β) estimated by linear mixed-effects models using the data in panel (a). Thick solid lines indicate significant responses relative to the pre-stimulus baseline (p_{FDR}<.05, corrected across trials and sites), while thin dotted lines indicate nonsignificant (*NS*) responses. **c)** Whole-cortex results overlaid on the participant's native cortical surface. Activation refers to HFB power significantly higher than baseline, while deactivation refers to HFB power significantly lower than baseline (p_{FDR}<.05, corrected across trials and sites). Mentalizing-active sites are colored by self/other selectivity, which was determined by robust regression of single-trial HFB peak power across mentalizing type (p_{FDR}<.05, corrected across sites). *Abbreviations***:** ROI = region-of-interest, Visual = visual cortex, ATL = anterior temporal lobe, TPJ = temporoparietal junction, PMC = posteromedial cortex, amPFC = anteromedial prefrontal cortex, dmPFC = dorsomedial prefrontal cortex, vmPFC = ventromedial prefrontal cortex.

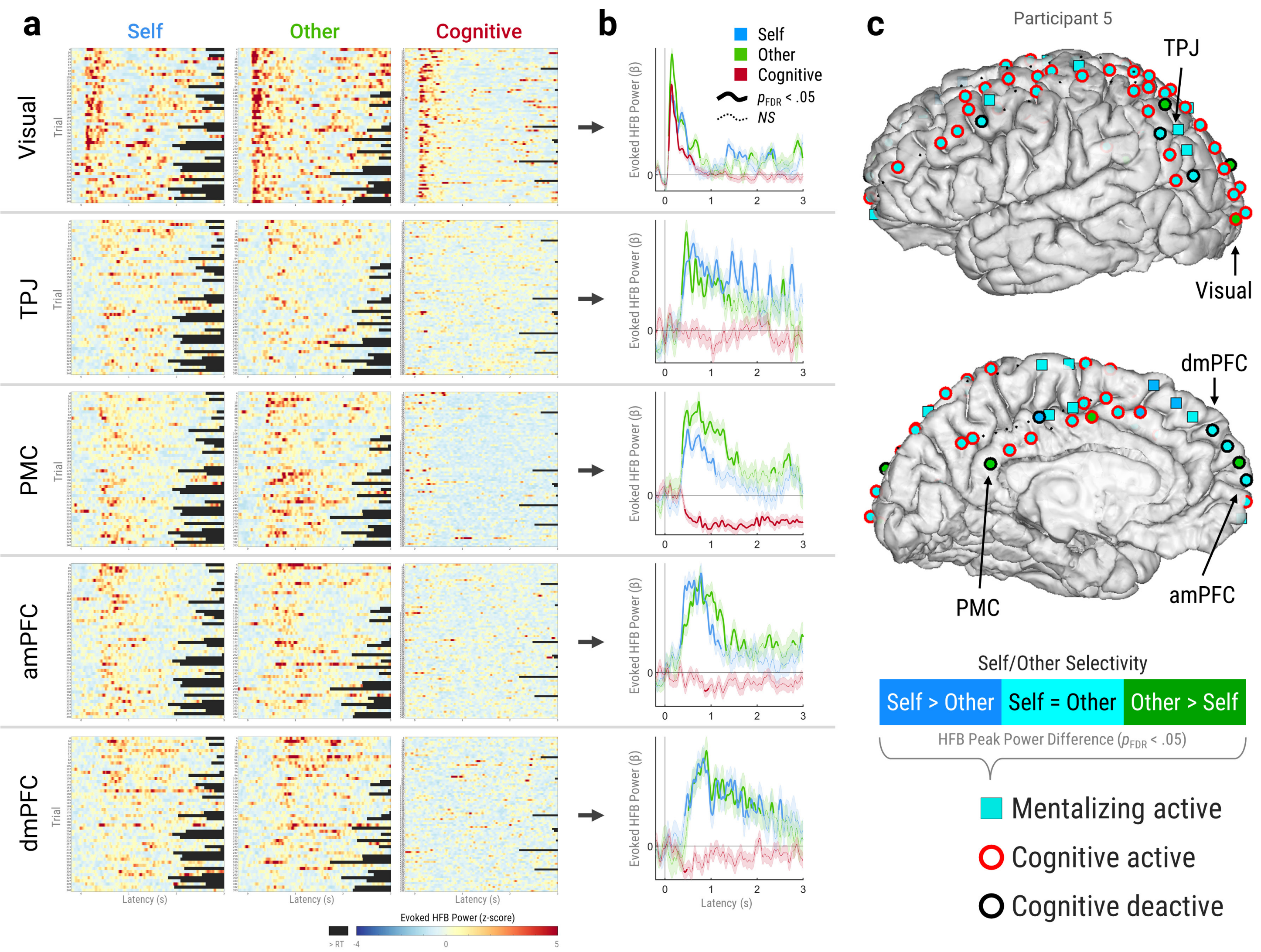

Supplementary Figure 3: Trialwise propagation of activation onsets across ROI groups (combined ROIs) during mentalizing. Dots indicate single-trial means from mentalizing-active ROI sites. Thin lines connect individual trials. Black lines connect grand means. Error bars indicate standard error of grand means. Colors indicate participant; color lightness slightly varied across trials to increase legibility. **a)** Participants with simultaneous coverage in two or more ROI groups. N=3558 (trials x ROI groups). **b)** Participants with simultaneous coverage in all ROI groups. N=2296 (trials x ROI groups). *Abbreviations*: ROI = region-of-interest, Visual = visual cortex, ATL = anterior temporal lobe, TPJ = temporoparietal junction, PMC = posteromedial cortex, amPFC = anteromedial prefrontal cortex, dmPFC = dorsomedial prefrontal cortex, vmPFC = ventromedial prefrontal cortex. Source data are provided as a Source Data file.

*ROI groups***:** Visual = visual cortex; tpDMN = temporoparietal DMN (TPJ, ATL & PMC); mPFC = medial prefrontal cortex (amPFC, dmPFC & vmPFC).

*Methods***:** To determine if onset latencies increased sequentially across ROI groups within trials (Visual < tpDMN < mPFC), we used random effects models with ROIgroup nested within Trial within Participant. This specification provides within-trial ROIgroup estimates while accounting for any missing ROI groups in certain participants in panel **a**. After model estimation, a trend contrast was performed on within-trial ROIgroup estimates [contrast coefficients = Visual(-3), tpDMN(1), mPFC(2)]. Heterogenous variance between trials, participants, and ROI groups was accounted for by unconstrained variance-covariance parameters between random effect nesting levels, along with Satterthwaite approximation for degrees of freedom. This analysis was performed separately using participants in panels **a** and **b**.

*Results***: a)** For participants with coverage in 2+ ROI groups, we found a robust within-trial trend of onset latency differences across ROIs ($F_{1,1538}$=3408, p<2.23e-308). **b)** For participants with coverage in all ROI groups, the trend contrast was also robust ($F_{1,862}$=2642, p=1.04e-264).

Conclusions: These results show consistent trial-by-trial propagation of mentalizing activations, which began in visual cortex, then spread to tpDMN regions, and finally to mPFC regions. Taken together, these results portray a pathway of mentalizing processing across ROIs.

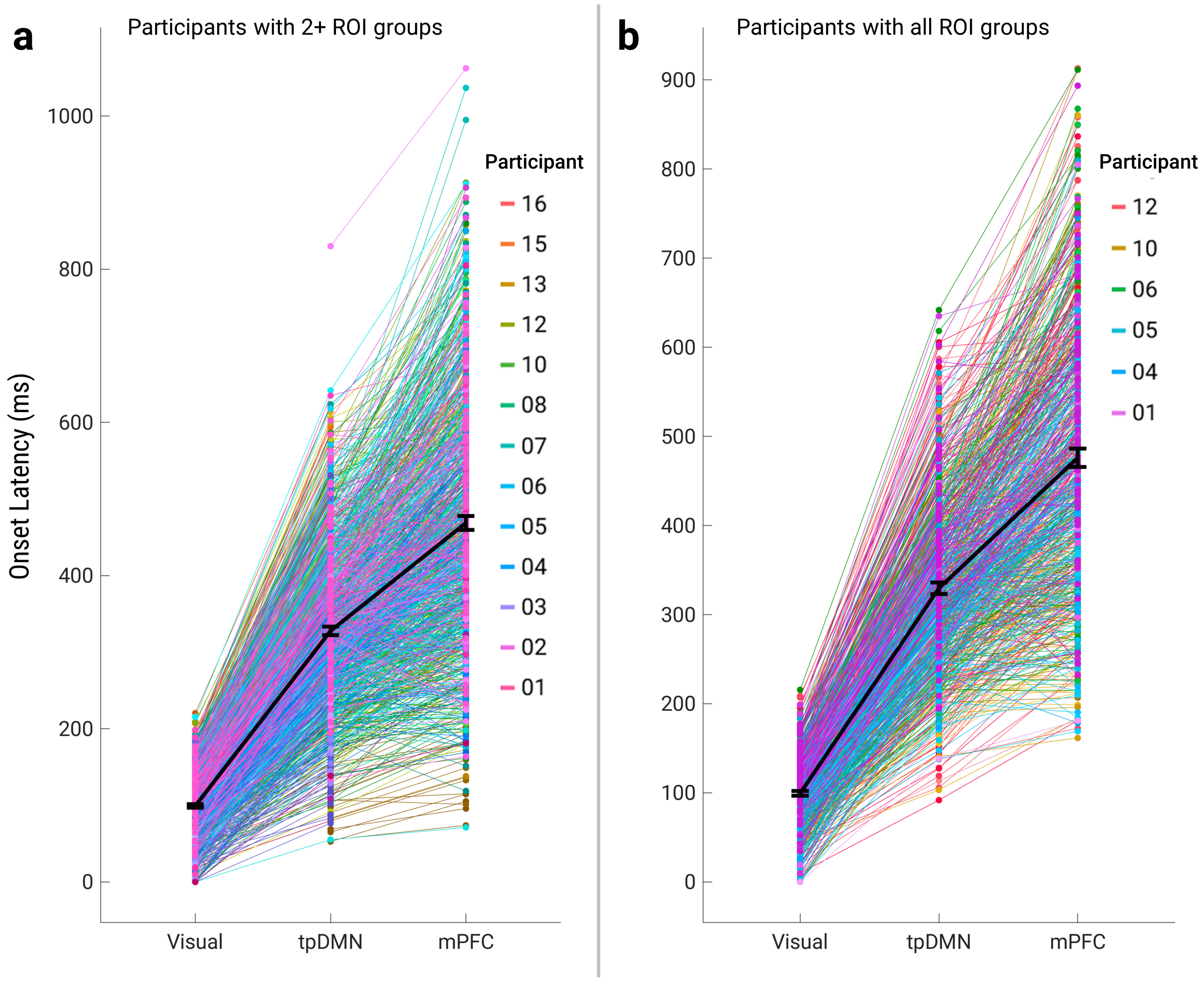

Supplementary Figure 4: Behavioral results. See corresponding section of Main Results for statistical results. Boxplots represent Q1 (25%), median (50%), and Q3 (75%) as the lower, center, and upper bounds of the box, respectively. Boxplot whisker minima indicate Q1-1.5*interquartile range. Boxplot whisker maxima indicate Q3+1.5*interquartile range. **a)** Distribution of behavioral response times (RT_{Behav}). The thick black line shows the upper 5000 ms cutoff for aggregate ROI analyses of mentalizing (see Table 1). *N*=3150 trials. **b)** Accuracy of cognitive task trials (arithmetic). Accuracy was not available for mentalizing trials due to the subjectiveness of trait judgments and participant-specific mentalizing targets (themselves and a previous or current neighbor). *N*=1400 trials. **c)** Behavioral response choices ($Choice_{Behav}$) across the affective valence of self- and other-mentalizing prompts. The bolded black stars indicate the grand mean across all participants. *N*=1475 trials. Source data are provided as a Source Data file.

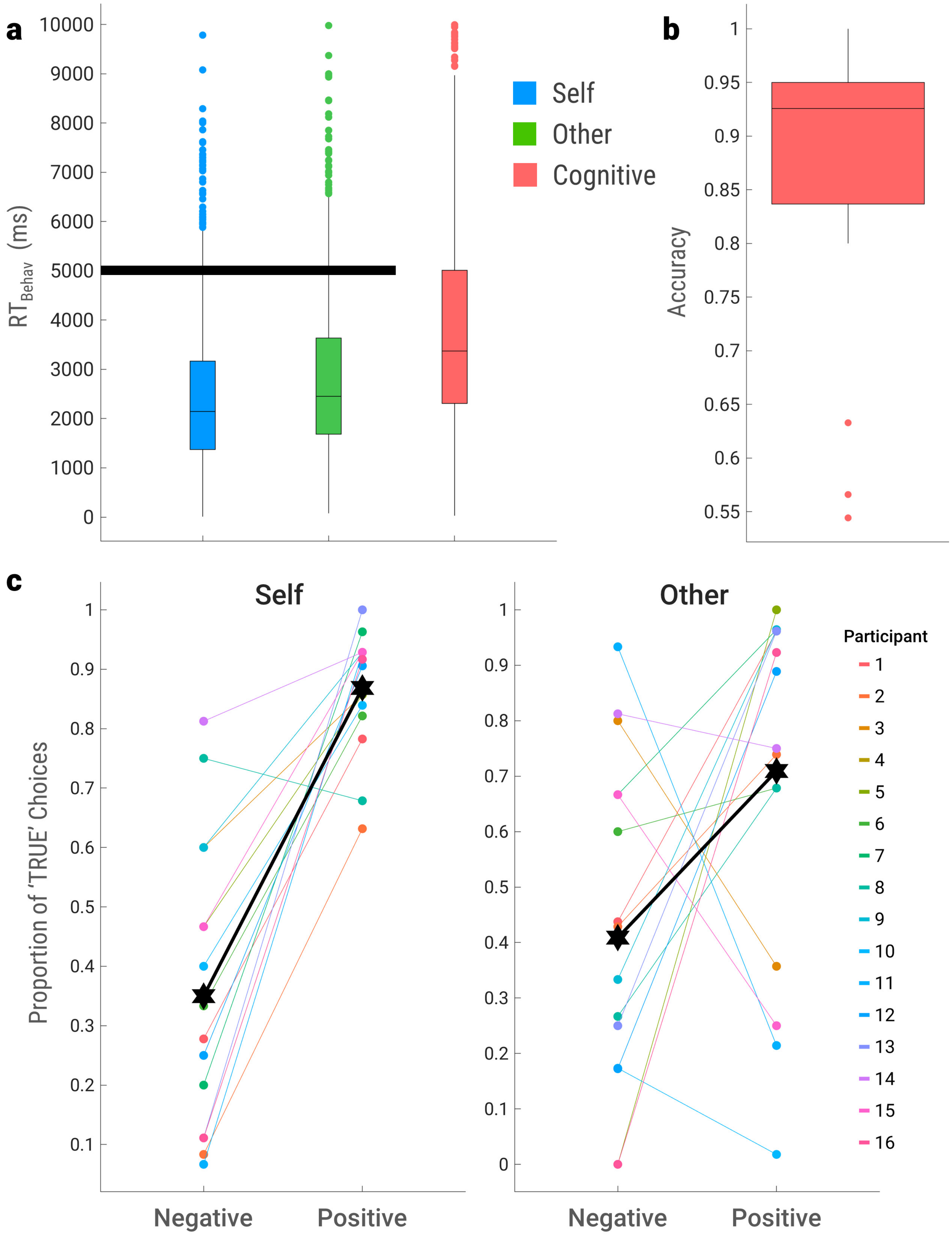

Supplementary Figure 5: Mean activation latencies within mentalizing type and ROIs using behavior-matched trials. Mean behavioral response time was 2000±5 ms for each ROI and mentalizing type (self-mentalizing = blue, other-mentalizing = green). Left and right floating bar edges represent onsets and offsets, respectively, while diamonds represent peaks (see Fig. 1e). Error bars indicate standard error of the mean. *N*=2124 (trials x sites). *Abbreviations*: ROI = region-of-interest, Vis = visual cortex, ATL = anterior temporal lobe, TPJ = temporoparietal junction, PMC = posteromedial cortex, amPFC = anteromedial prefrontal cortex, dmPFC = dorsomedial prefrontal cortex, vmPFC = ventromedial prefrontal cortex. Source data are provided as a Source Data file.

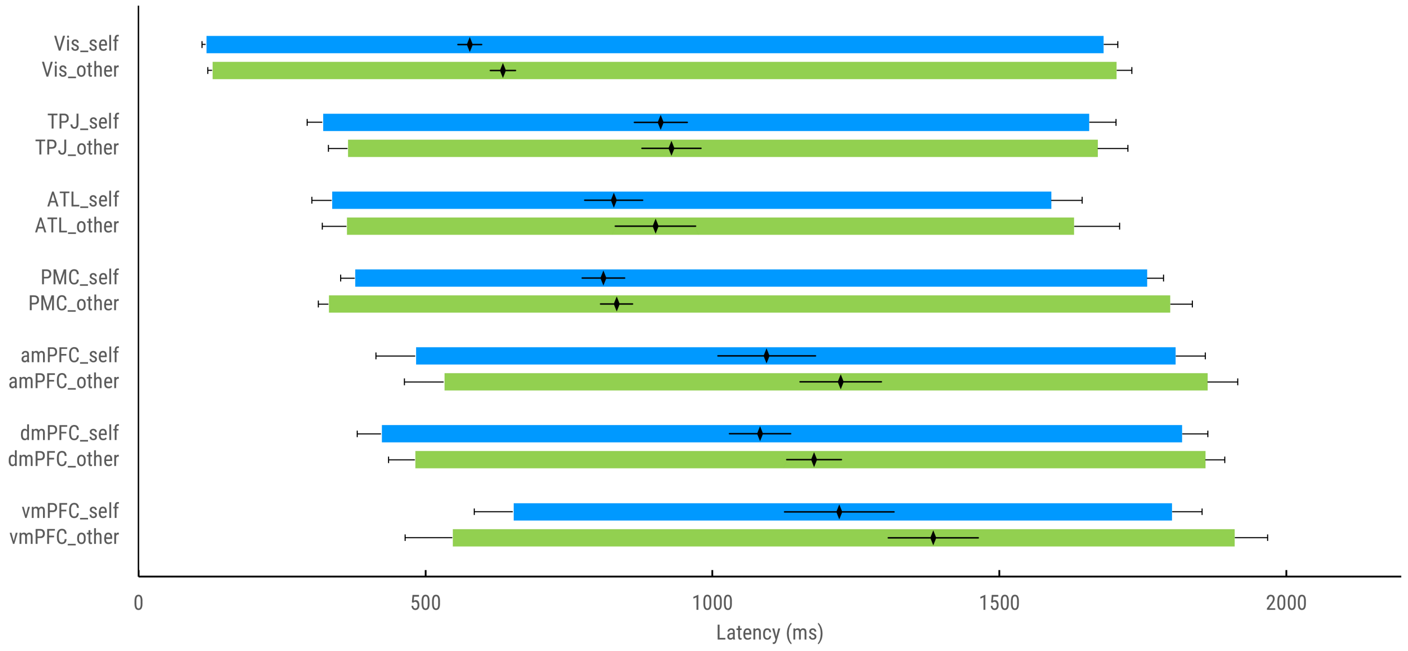

Supplementary Figure 6: Expanded lateralized views of Fig. 3a. Sites identified as active, deactive, or nonresponsive for mentalizing versus baseline (p_{FDR}<.05, corrected across timepoints and sites). Left hemisphere on left column, right hemisphere on right column. *Abbreviations*: Mz = mentalizing (collapsed across self and other).

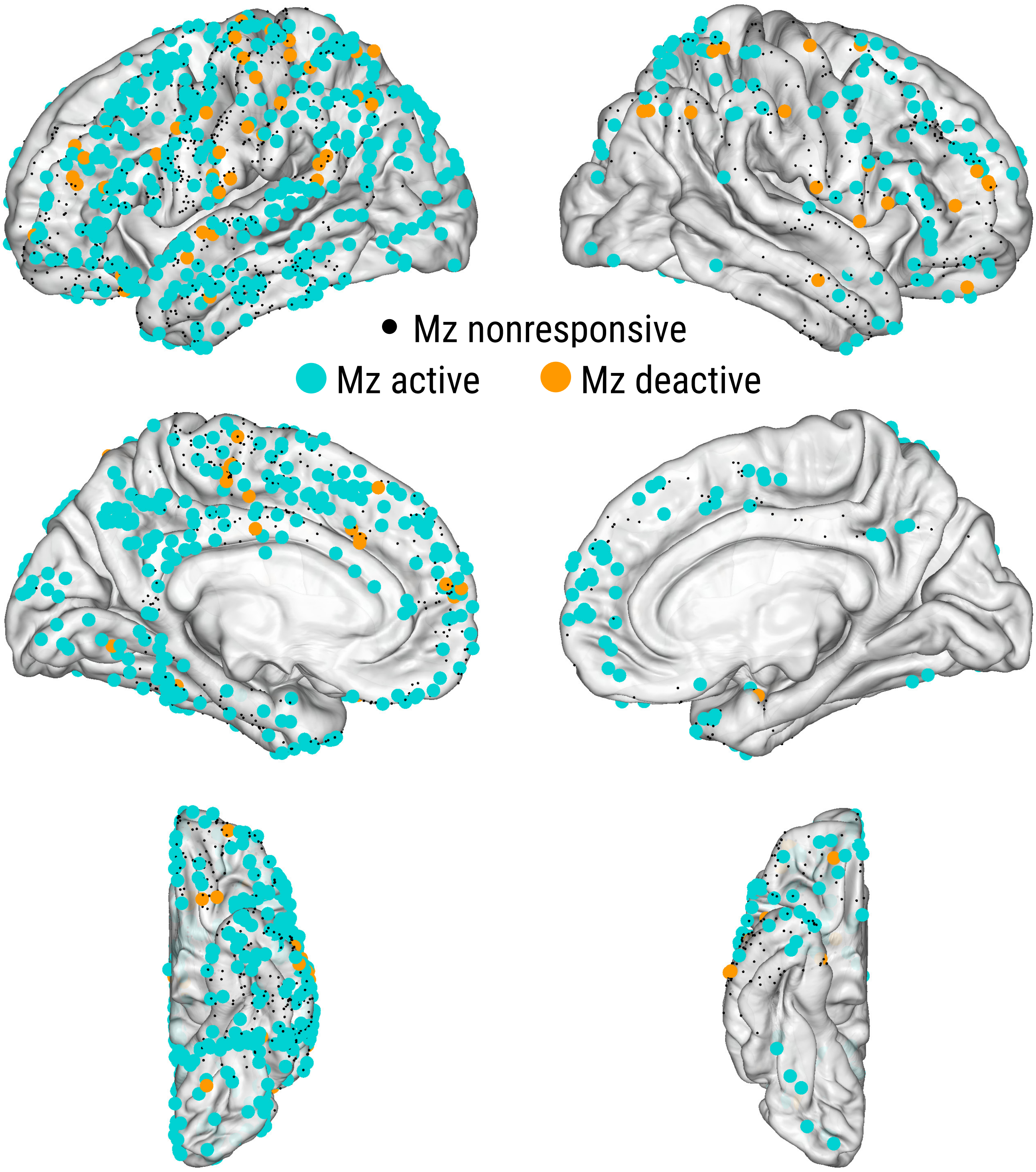

Supplementary Figure 7: Expanded lateralized views of Fig. 3c. Functional specificity of mentalizing-active sites. Functional specificity of mentalizing-active sites. †Mentalizing-specific sites, defined as mentalizing-active but not cognitive-active (p_{FDR}<.05, corrected across timepoints and sites) with significantly higher peak power for mentalizing (p_{FDR}<.05, corrected across sites). §Non-specific sites, defined as mentalizing-active and cognitive-active. Left hemisphere on left column, right hemisphere on right column. *Abbreviations*: Mz = mentalizing (collapsed across self and other), Cog = cognitive task (arithmetic).

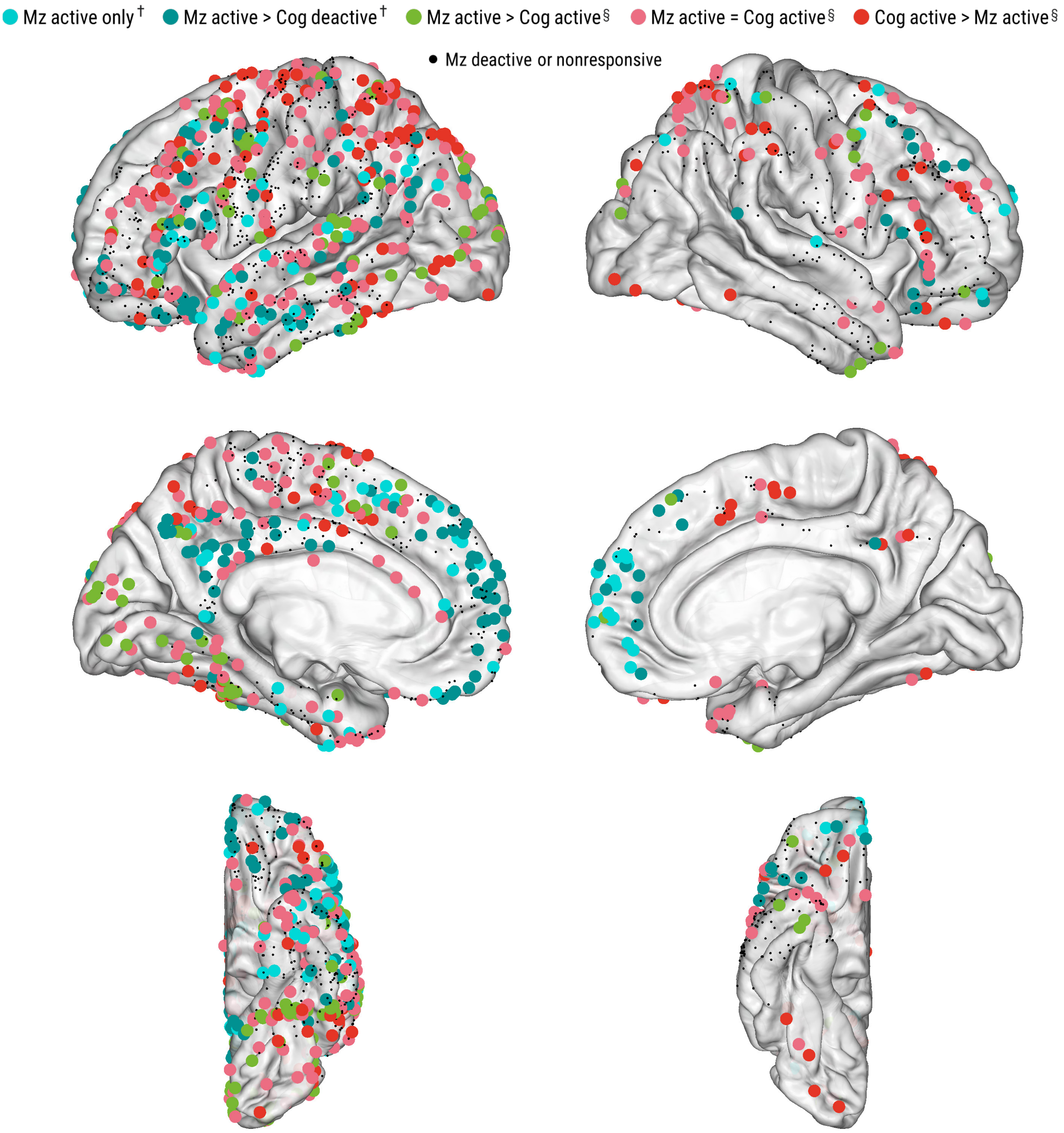

Supplementary Figure 8: Expanded lateralized views of Fig. 4a. Mean onset latencies of mentalizing-active sites (p_{FDR}<.05, corrected across timepoints and sites). Left hemisphere on left column, right hemisphere on right column.

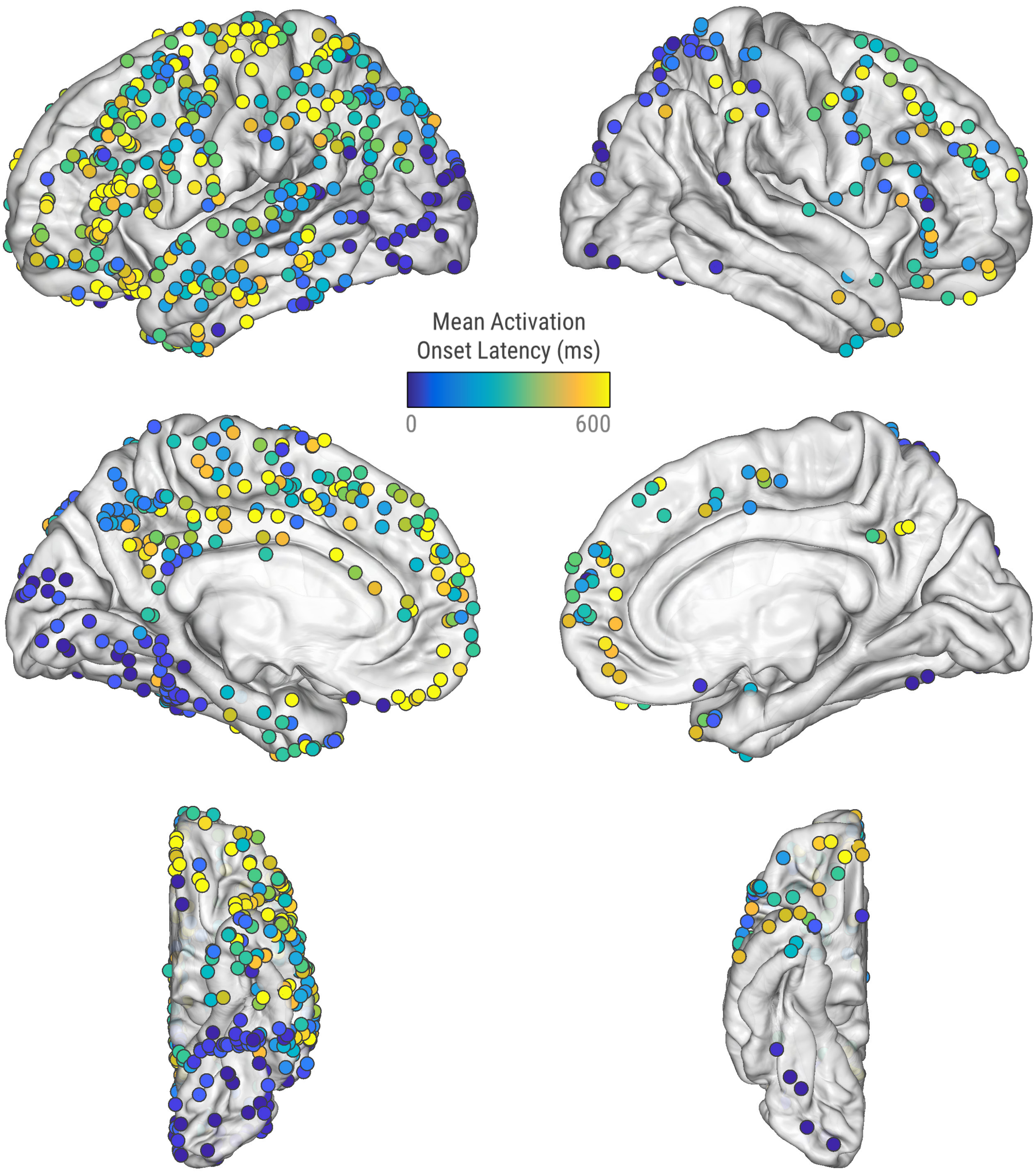

Supplementary Figure 9: Expanded lateralized views of Fig. 5a. Functional anatomy of self- and other-mentalizing. Circles represent sites identified as coactive for both mentalizing types versus baseline (p_{FDR}<.05, corrected across timepoints and sites), colored by self/other differences in peak power (p_{FDR}<.05, corrected across sites; see Fig. 2). Squares represent sites identified as active for only one mentalizing type with significantly higher peak power for that mentalizing type. Black dots represent sites with nonsignificant mentalizing activations. Left hemisphere on left column, right hemisphere on right column.

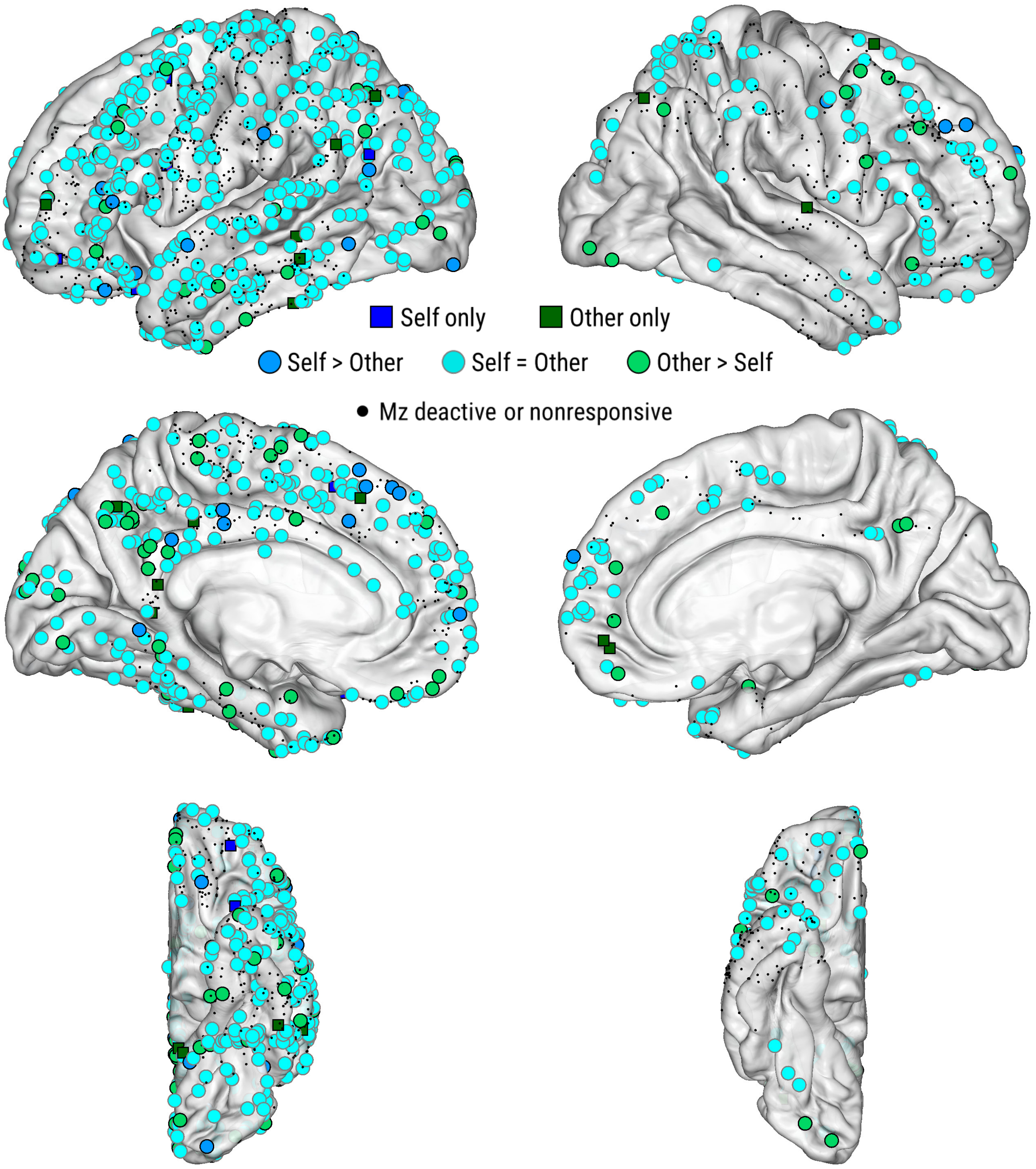

Supplementary Table 2: Participant demographics and characteristics.

Participant ECoG implant hemisphere: 12 left, 4 right
Participant handedness: 1 left, 13 right, 2 ambidextrous
Participant IQ: median = 93, range = 71-119

Participant	Age	Sex
S1	62	F
S2	40	M
S3	24	F
S4	36	F
S5	22	F
S6	51	F
S7	46	M
S8	31	F
S9	52	F
S10	28	M
S11	33	M
S12	23	F
S13	38	F
S14	44	M
S15	22	M
S16	48	F

Supplementary Table 3: Summary statistics of single-trial HFB metrics from mentalizing-active ROI sites. Source data are provided as a Source Data file. Abbreviations: HFB = high-frequency broadband (70-180 Hz), *N* = number of participants; L = number of sites in left hemisphere; R = number of sites in right hemisphere; *n* = number of sites with significant (p_{FDR}<.05) trial-averaged HFB activations; ms = millisecond; *M* = sample mean; *SE* = standard error of the mean

						**Latency** (ms)						**Duration** (ms)		**Peak Power** (z-score)	
						**Onset**		**Peak**		**Offset**					
**ROI**	*N*	L	R	**Condition**	*n*	*M*	*SE*	*M*	*SE*	*M*	*SE*	*M*	*SE*	*M*	*SE*
Visual	14	95	7	Self	71	99	2	514	6	2254	19	969	12	2.067	0.013
				Other	71	102	2	594	7	2273	19	1002	11	2.389	0.017
TPJ	14	76	19	Self	34	304	9	1044	20	2156	31	668	15	1.629	0.018
				Other	36	308	9	1084	21	2225	31	713	16	1.758	0.017
ATL	12	74	38	Self	37	311	9	957	18	2030	32	545	12	1.454	0.015
				Other	37	328	11	1133	22	2429	31	642	14	1.560	0.016
PMC	10	66	9	Self	46	335	7	855	12	2058	26	734	13	1.696	0.017
				Other	50	328	7	929	13	2446	25	894	15	1.910	0.019
amPFC	11	36	13	Self	22	446	14	1197	25	2266	42	703	18	1.660	0.019
				Other	23	482	15	1438	30	2622	40	852	21	1.752	0.021
dmPFC	10	54	27	Self	36	452	10	1116	19	2325	32	716	13	1.678	0.018
				Other	36	483	11	1419	21	2623	31	838	16	1.691	0.017
vmPFC	11	33	8	Self	10	537	22	1382	33	2444	47	734	24	1.816	0.028
				Other	11	539	21	1679	36	3033	43	1148	36	2.143	0.032

Supplementary Table 4: Examples of stimulus prompts used in our behavioral task (see Fig. 1a for task).

Self-mentalizing	Other-mentalizing	Cognitive (arithmetic)
I am caring	My neighbor is caring	1 + 18 = 19
I am confident	My neighbor is confident	1 + 18 = 25
I am curious	My neighbor is curious	1 + 41 = 42
I am demanding	My neighbor is demanding	1 + 41 = 50
I am focused	My neighbor is focused	10 + 1 = 11
I am a good listener	My neighbor is a good listener	10 + 1 = 17
I am happy	My neighbor is happy	11 + 2 = 13
I am loving	My neighbor is loving	11 + 2 = 16
I am moody	My neighbor is moody	16 + 8 = 22
I am nurturing	My neighbor is nurturing	16 + 8 = 24
I am patient	My neighbor is patient	2 + 52 = 54
I am a quick learner	My neighbor is a quick learner	2 + 52 = 56
I am quiet	My neighbor is quiet	2 + 60 = 62
I am relaxed	My neighbor is relaxed	2 + 60 = 72
I am rude	My neighbor is rude	20 + 4 = 17
I am selfish	My neighbor is selfish	20 + 4 = 24
I am serious	My neighbor is serious	24 + 2 = 19
I am silly	My neighbor is silly	24 + 2 = 26
I am easily agitated	My neighbor is easily agitated	24 + 5 = 29
I am emotional	My neighbor is emotional	24 + 5 = 39
I am compassionate	My neighbor is compassionate	24 + 7 = 31
I am competitive	My neighbor is competitive	24 + 7 = 35
I am controlling	My neighbor is controlling	26 + 7 = 33
I am creative	My neighbor is creative	26 + 7 = 34
I am dependable	My neighbor is dependable	28 + 6 = 34
I am easily bored	My neighbor is easily bored	28 + 6 = 32
I am easily disappointed	My neighbor is easily disappointed	33 + 5 = 32
I am easily distracted	My neighbor is easily distracted	33 + 5 = 38
I am easily frustrated	My neighbor is easily frustrated	38 + 6 = 42
I am easily scared	My neighbor is easily scared	38 + 6 = 44
I am easily stressed	My neighbor is easily stressed	4 + 25 = 29
I am easily upset	My neighbor is easily upset	4 + 25 = 32
I am energetic	My neighbor is energetic	42 + 6 = 48
I am friendly	My neighbor is friendly	42 + 6 = 59
I am funny	My neighbor is funny	44 + 9 = 51
I am generous	My neighbor is generous	44 + 9 = 53
I am hard-working	My neighbor is hard-working	45 + 9 = 54
I am helpful	My neighbor is helpful	45 + 9 = 50
I am honest	My neighbor is honest	47 + 8 = 54
I am kind	My neighbor is kind	47 + 8 = 55
I am lazy	My neighbor is lazy	47 + 9 = 56
I am outgoing	My neighbor is outgoing	47 + 9 = 56
I am polite	My neighbor is polite	5 + 63 = 68
I am respectful	My neighbor is respectful	5 + 63 = 56
I am sensitive	My neighbor is sensitive	5 + 87 = 92
I am shy	My neighbor is shy	5 + 87 = 90
I am smart	My neighbor is smart	9 + 21 = 30
I am talkative	My neighbor is talkative	9 + 21 = 33
I am observant	My neighbor is observant	49 + 8 = 57
I am thoughtful	My neighbor is thoughtful	49 + 8 = 52

Supplementary References

1. Jarrett, K., Kavukcuoglu, K., Ranzato, M. & LeCun, Y. What is the best multi-stage architecture for object recognition? in *2009 IEEE 12th International Conference on Computer Vision* 2146–2153 (2009). doi:10.1109/ICCV.2009.5459469.
2. Leeds, D. D., Seibert, D. A., Pyles, J. A. & Tarr, M. J. Comparing visual representations across human fMRI and computational vision. *Journal of vision* **13**, 25–25 (2013).
3. Kruskal, J. B. Multidimensional scaling by optimizing goodness of fit to a nonmetric hypothesis. *Psychometrika* **29**, 1–27 (1964).
4. Victoria, L. W., Pyles, J. A. & Tarr, M. J. The relative contributions of visual and semantic information in the neural representation of object categories. *Brain Behav* **9**, e01373 (2019).
5. Yang, Y., Tarr, M. J., Kass, R. E. & Aminoff, E. M. Exploring spatiotemporal neural dynamics of the human visual cortex. *Human Brain Mapping* **40**, 4213–4238 (2019).
6. Popham, S. F. *et al.* Visual and linguistic semantic representations are aligned at the border of human visual cortex. *Nat Neurosci* **24**, 1628–1636 (2021).
