# Supplementary material for: Electrocorticographic evidence of a common neurocognitive sequence for mentalizing about the self and others
Source: Nat Commun. 2022 Apr 8;13:1919. doi: 10.1038/s41467-022-29510-2 (PMC8993891; doi:10.1038/s41467-022-29510-2)
Supplement: Supplementary file 3 — Reporting Summary [file 41467_2022_29510_MOESM3_ESM.pdf]

Corresponding author(s): Matthew D. Lieberman

Last updated by author(s): Jan 11, 2022

Reporting Summary

Nature Portfolio wishes to improve the reproducibility of the work that we publish. This form provides structure for consistency and transparency in reporting. For further information on Nature Portfolio policies, see our Editorial Policies and the Editorial Policy Checklist.

Statistics

For all statistical analyses, confirm that the following items are present in the figure legend, table legend, main text, or Methods section.

n/a	Confirmed	
☐	☒	The exact sample size (*n*) for each experimental group/condition, given as a discrete number and unit of measurement
☐	☒	A statement on whether measurements were taken from distinct samples or whether the same sample was measured repeatedly
☐	☒	The statistical test(s) used AND whether they are one- or two-sided<br>*Only common tests should be described solely by name; describe more complex techniques in the Methods section.*
☐	☒	A description of all covariates tested
☐	☒	A description of any assumptions or corrections, such as tests of normality and adjustment for multiple comparisons
☐	☒	A full description of the statistical parameters including central tendency (e.g. means) or other basic estimates (e.g. regression coefficient) AND variation (e.g. standard deviation) or associated estimates of uncertainty (e.g. confidence intervals)
☐	☒	For null hypothesis testing, the test statistic (e.g. *F*, *t*, *r*) with confidence intervals, effect sizes, degrees of freedom and *P* value noted<br>*Give P values as exact values whenever suitable.*
☒	☐	For Bayesian analysis, information on the choice of priors and Markov chain Monte Carlo settings
☐	☒	For hierarchical and complex designs, identification of the appropriate level for tests and full reporting of outcomes
☐	☒	Estimates of effect sizes (e.g. Cohen's *d*, Pearson's *r*), indicating how they were calculated

Our web collection on statistics for biologists contains articles on many of the points above.

Software and code

Policy information about availability of computer code

Data collection	MATLAB 2020b, Psychophysics Toolbox 3, BioImage Suite v1.2
Data analysis	MATLAB 2020b; MATLAB Statistics and Machine Learning Toolbox 2020b (linear mixed-effects models, logistic mixed-effects classification, bisquare robust regression, Welch's t-tests); Freesurfer v7; BioImage Suite v1.2; iELVis Toolbox v1.1; custom code available here: https://github.com/pinheirochagas/lbcn_preproc

For manuscripts utilizing custom algorithms or software that are central to the research but not yet described in published literature, software must be made available to editors and reviewers. We strongly encourage code deposition in a community repository (e.g. GitHub). See the Nature Portfolio guidelines for submitting code & software for further information.

Data

Policy information about availability of data

All manuscripts must include a data availability statement. This statement should provide the following information, where applicable:

- Accession codes, unique identifiers, or web links for publicly available datasets
- A description of any restrictions on data availability
- For clinical datasets or third party data, please ensure that the statement adheres to our policy

Source data are provided with this paper. The behavioral dataset is provided as source data for Supplementary Fig. 4. Anonymized preprocessed ECoG data can be shared upon reasonable request, subject to a data-sharing agreement between the requestor(s), study authors, and Stanford University. The data-sharing agreement will be tailored to the aims of the requestor(s). Contact Kevin M. Tan: kevmtan@ucla.edu.

Field-specific reporting

Please select the one below that is the best fit for your research. If you are not sure, read the appropriate sections before making your selection.

- [x] Life sciences
- [ ] Behavioural & social sciences
- [ ] Ecological, evolutionary & environmental sciences

For a reference copy of the document with all sections, see nature.com/documents/nr-reporting-summary-flat.pdf

Life sciences study design

All studies must disclose on these points even when the disclosure is negative.

Sample size	Participants consisted of 16 neurosurgical patients who were implanted with electrocorticography (ECoG) for epilepsy monitoring and treatment (10 female, 6 male; 22-62 years old). Patients were included in this subject's cohort if they had ECoG coverage in a priori default network regions. A priori sample size calculations were not performed due to the rarity of ECoG data; inclusion in the current sample was constrained by patient clinical needs. The current sample size is larger than most ECoG studies and produced sufficient fit in our statistical analyses. The current sample is not meant to be representative of any particular demographics.
Data exclusions	Electrode sites were discarded from further analyses if they were marked as pathological or 'noisy' by postclinical evaluation using pre-established clinical criteria.
Replication	Attempts are replicating these results were not performed due to the inherent difficulty and invasiveness of ECoG acquisition. Moreover, each participant had unique placement of ECoG electrodes according to their specific clinical needs. Participants no longer have ECoG implants thus the study cannot be replicated using our cohort.
Randomization	This study did not involve experimental groups; each subject underwent the same experimental paradigm.
Blinding	No blinding was performed as all participants underwent the same experimental protocol; participants were not divided into groups or treatments.

Reporting for specific materials, systems and methods

We require information from authors about some types of materials, experimental systems and methods used in many studies. Here, indicate whether each material, system or method listed is relevant to your study. If you are not sure if a list item applies to your research, read the appropriate section before selecting a response.

Materials & experimental systems

n/a	Involved in the study	
x		Antibodies
x		Eukaryotic cell lines
x		Palaeontology and archaeology
x		Animals and other organisms
	x	Human research participants
x		Clinical data
x		Dual use research of concern

Methods

n/a	Involved in the study	
x		ChIP-seq
x		Flow cytometry
	x	MRI-based neuroimaging

Human research participants

Policy information about studies involving human research participants

Population characteristics	Participants consisted of 16 neurosurgical patients who were implanted with electrocorticography (ECoG) for epilepsy monitoring and treatment (10 female, 6 male; 22-62 years old). Patients were included in this subject's cohort if they had ECoG coverage in default network regions. This sample is not meant to be representative of any particular demographics.
Recruitment	Neurosurgical epileptic patients were included in this subject's cohort if they had ECoG coverage in default network regions. Sites and timepoints that exhibited epileptic activity were excluded, which should mitigate confounds across epileptic and healthy populations (see Parvizi & Kastner, 2019), though such confounds cannot be fully ameliorated.
Ethics oversight	Stanford Institutional Review Board

Note that full information on the approval of the study protocol must also be provided in the manuscript.

Magnetic resonance imaging

Experimental design

Design type	Magnetic resonance imaging was used for structural data only; event-related design for electrocorticography
Design specifications	Two runs per participant, each consisting of 50-80 trials per experimental condition. Trials lasted until a behavioral response or up to 15 seconds if no response. Trials were separated by a 200 ms inter-trial interval consisting of fixation crosshair.
Behavioral performance measures	Button presses and response times were collected. Trials with irrelevant or missing button presses were excluded. Trials with response times under 400 ms were excluded. 'Cognitive task' trials had median accuracy of 92.5%. No accuracy metrics for mentalizing trials due to subjectiveness of task. To confirm that participants attentively performed the mentalizing task, we show that behavioral response choices had differential self/other biases towards positive or negative affective traits, indicating that participants discerned the targets and traits of mentalizing prompts.

Acquisition

Imaging type(s)	Structural
Field strength	Three Tesla
Sequence & imaging parameters	GE 3-Tesla SIGNA Magnetic Resonance Imaging (MRI) scanner at Stanford University. A T1-weighted anterior-posterior commissure-aligned pulse sequence was used. T1 data was resampled to 1 mm isotropic voxels, then segmented to distinguish gray and white matter using FreeSurfer.
Area of acquisition	Whole Brain

Diffusion MRI ☐ Used ☒ Not used

Preprocessing

Preprocessing software	Freesurfer v7.1
Normalization	Surface-based normalization
Normalization template	Freesurfer average template, MNI305 template
Noise and artifact removal	Freesurfer structural segmentation
Volume censoring	N/A; structural scan only

Statistical modeling & inference

Model type and settings	N/A; structural scan only
Effect(s) tested	N/A; structural scan only

Specify type of analysis: ☒ Whole brain ☐ ROI-based ☐ Both

Statistic type for inference (See Eklund et al. 2016)	N/A; structural scan only
Correction	N/A; structural scan only

Models & analysis

n/a	Involved in the study
☒	☐ Functional and/or effective connectivity
☒	☐ Graph analysis
☒	☐ Multivariate modeling or predictive analysis
